# Clinical Characteristics and Long-Term Recombinant Human Growth Hormone Treatment of 18q- Syndrome: A Case Report and Literature Review

**DOI:** 10.3389/fendo.2021.776835

**Published:** 2021-12-09

**Authors:** Shanshan Liu, Meiping Chen, Hongbo Yang, Shi Chen, Linjie Wang, Lian Duan, Huijuan Zhu, Hui Pan

**Affiliations:** Key Laboratory of Endocrinology of National Health Commission, Department of Endocrinology, State Key Laboratory of Complex Severe and Rare Diseases Peking Union Medical College Hospital, Chinese Academy of Medical Science and Peking Union Medical College, Beijing, China

**Keywords:** 18q- syndrome, clinical characteristic, short stature, growth hormone deficiency, rhGH treatment

## Abstract

**Background:**

18q- syndrome is a rare chromosomal disease caused by the deletion of the long arm of chromosome 18. Some cases with 18q- syndrome can be combined with growth hormone deficiency (GHD), but data on the efficacy of recombinant human growth hormone (rhGH) treatment in 18q- syndrome are limited.

**Methods:**

Here, we report one case of 18q- syndrome successfully treated with long-term rhGH supplement. Previously reported cases in the literature are also reviewed to investigate the karyotype–phenotype relationship and their therapeutic response to rhGH.

**Results:**

A 7.9-year-old girl was referred for evaluation for short stature. Physical exam revealed proportionally short stature with a height of 111.10 cm (−3.02 SD score (SDS)), low-set ears, a high-arched palate, a small jaw, webbed neck, widely spaced nipples, long and tapering fingers, and cubitus valgus. Thyroid function test indicated subclinical hypothyroidism. The peak value of growth hormone was 10.26 ng/ml in the levodopa provocation test. Insulin-like growth factor 1 (IGF-1) was 126 ng/ml (57–316 ng/ml). Other laboratory investigations, including complete blood cell count, liver and kidney function, gonadal function, serum adrenocorticotropin levels, and serum cortisol levels, were all within normal ranges. Karyotype analysis showed 46, XX, del (18) (q21). L-Thyroxine replacement and rhGH treatment were initiated and maintained in the following 7 years. At the age of 14.8, her height has reached 159.5 cm with a height SDS increase of 2.82 SDS (from −3.02 SDS to −0.20 SDS). No significant side effects were found during the treatment. The literature review indicated the average rhGH treatment duration of 16 patients was 5.9 ± 3.3 years, and the average height SDS significantly increased from −3.12 ± 0.94 SDS to −1.38 ± 1.29 SDS after the rhGH treatment (p < 0.0001).

**Conclusion:**

The main clinical manifestations of 18q- syndrome include characteristic appearance, intellectual disability, and abnormal genital development. The literature review suggested a significant height benefit for short stature with 18q- syndrome from long-term rhGH treatment.

## Introduction

Chromosome 18q- syndrome (OMIM# 601808) is a rare chromosomal disease, which was first reported by De Grouchy in 1964, and more than 100 cases had been reported to date (1). Previous studies suggested a distal critical region located in 18q22.3–23 (67.7–74.9 basis) and a proximal critical region located in 18q12.1–q12.3 (25.2–42.9 basis) (2). The most common cause of the syndrome is the deletion of the terminal of the long arm of chromosome 18 (3). The clinical phenotypes of 18q- syndrome are highly variable due to heterogeneity with variable size and genetic content and imbalances from structurally abnormal chromosomes. Commonly reported phenotypic features of 18q- syndrome are as follows: 1) physical deformities including mid-face dysplasia, ear canal deformity, hand or foot abnormalities, and genital dysplasia; 2) psychoneurological abnormalities including intellectual disability, hypotonia, language and motor development delay, and hearing impairment; 3) and short stature with or without growth hormone deficiency (GHD) (4). Short stature is a common and important issue in patients with 18q- syndrome, and concurrence of GHD had been reported in some of these patients (5–7). However, information about the efficacy of recombinant human growth hormone (rhGH) treatment in patients with 18q- syndrome is limited.

Here, we report a case with 18q- syndrome who was successfully treated with long-term rhGH supplement. Previously reported cases in the literature are also reviewed to investigate the karyotype–phenotype relationship and their therapeutic response to rhGH.

## Materials and Methods

### Participant

In this study, the patient underwent physical examination and laboratory investigations for evaluation of short stature. Complete blood cell count, liver and kidney function, thyroid function, gonadal function, serum adrenocorticotropin levels and serum cortisol levels, serum insulin-like growth factor-1 (IGF-1) levels, and GH stimulation tests were all tested in the central laboratory of Peking Union Medical College Hospital. In the levodopa growth hormone provocation test, changes in GH levels were monitored in blood samples collected before medication and 30, 60, 90, and 120 min following levodopa administration. Peak GH of less than 10 ng/ml was adopted for the diagnosis of pediatric GHD in this study. Bone age was assessed using the G&P and TW3 methods (8).

Clinical information on the first and follow-up visits was collected, including birth history, history of growth and development, physical examination, laboratory, and radiological data.

### Literature Review

We searched for all articles in English on 18q- syndrome published up to May 2021, in which diagnosis of 18q- syndrome was confirmed by karyotype and clinical characters were described. Duplicated cases from the same research group were excluded. Clinical data, karyotypes, and rhGH treatment were recorded and summarized.

### Statistical Analysis

Statistical analysis was performed using SPSS.25 software. Continuous variables were expressed as the mean ± SD statement. A paired-samples t-test was used to compare the variables before and after the rhGH treatment. A p-value of less than 0.05 was considered statistically significant.

## Results

### Case Present

A 7.9-year-old girl was referred to our clinic for evaluation of short stature. She was the first child of healthy, non-consanguineous parents. She was born at term by cesarean section because of breech presentation. Her birth weight was 2.75 kg (−2.00 SD to −1.00 SD), and her birth length was 46 cm (<−2.00 SD). The height of her father and mother were 177 cm (+0.71 SD score (SDS)) and 163 cm (+0.45 SDS), respectively, with a mid-parental height (MPH) of 163.5 cm. She presented abnormal sucking at birth and mild intellectual disability from childhood. Due to poor coordination and inattention, artificial feeding was performed up to 4 years old.

Physical examination at the first evaluation revealed her height was 111.1 cm (−3.02 SDS) and weight was 19 kg (−1.46 SDS). She had low-set ears, a high-arched palate, a small jaw, webbed neck, widely spaced nipples, long and tapering fingers, and cubitus valgus. The thyroid gland was non-palpable. The peak value of growth hormone was 10.26 ng/ml in the levodopa growth hormone provocation test. IGF-1 was 126 ng/ml (57–316 ng/ml). Thyrotropin (TSH) level was 6.62 μIU/ml (0.38–4.34), free triiodothyronine (FT3) was 4.10 pg/ml (1.80–4.10), and free thyroxine (FT4) was 1.51 ng/dl (0.81–1.89). Anti-thyroid peroxidase and anti-thyroglobulin levels were all in normal ranges. Other laboratory investigations, including complete blood cell count, liver and kidney function, gonadal function, serum adrenocorticotropin levels, and serum cortisol levels, were all in normal ranges. The initial bone age was consistent with chronological age. Karyotype analysis showed 46, XX, del (18) (q21).

**Figure 1:**
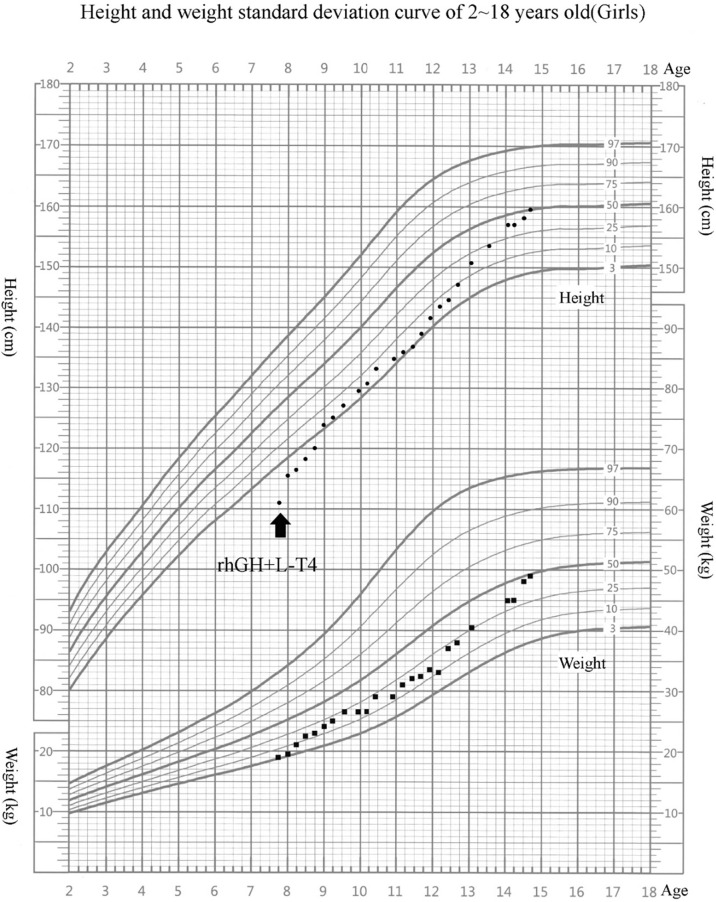
Growth chart of our patient. Arrow indicates the inception of recombinant human growth hormone (rhGH) treatment. The girl’s growth curve significantly improved with GH treatment over time. Height curve, circles; weight curve, squares.

L-Thyroxine (L-T4) replacement therapy and rhGH treatment were initiated. rhGH was started at 0.05 mg/kg/day and titrated gradually during follow-up, with an average dose of about 0.06 mg/kg/day. She has been treated for 7 years, and her compliance was good. Her height reached 159.5 cm (−0.20 SDS) at 14 years 8 months. During rhGH treatment, the growth velocity (GV) was 8.3 cm/year in the first year and 7.8 cm/year in the second year (**Figure 1**). Bone age was consistent with chronological age. She presented with normal onset of puberty and menarche at 14 years. There were no serious adverse reactions during long-term rhGH treatment.

### Phenotype Summaries of 18q- Syndrome in Literature Review

**Figure 2:**
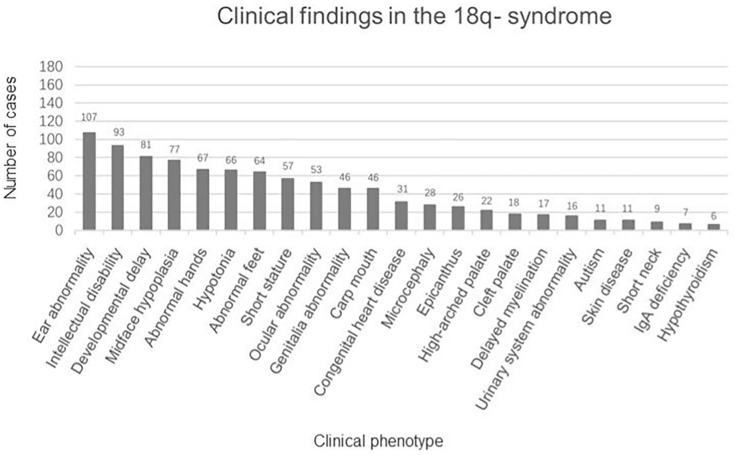
Clinical findings in the 163 patients with 18q- syndrome.

There were 162 eligible cases of 18q- syndrome enrolled in the study. Including the present case, a total of 163 cases were recorded and summarized. The median age was 5.75 years (ranging from 2.90 to 12.06 years), the average height SDS was −2.04 ± 1.36, and the average weight SDS was −1.01 ± 1.68. The clinical manifestations are summarized in **Figure 2**. The main features include the following: 1) physical deformities including ear abnormality (ear canal stenosis, hearing impairment, prominent antihelix, and antitragus) (65.6%), mid-face dysplasia (47.2%), abnormal hands (aschistodactylia, tapering fingers, clinodactylism, proximally implanted thumbs, excess whorls, and “simian-variant” palmar creases) (41.1%), abnormal feet (varus deformity, dactylion, and abnormal toes) (39.3%), short stature (35.0%), ocular abnormality (strabismus, corneal opacification, cataracts, glaucoma, optic atrophy, and nystagmus) (32.5%), abnormal genital development (blind uterine horns, uterine remnant, and absence of vagina/cervix) (28.2%), carp mouth (28.2%), congenital heart disease (19.0%), microcephaly (17.2%), epicanthus (16.0%), a high-arched palate (13.5%), a cleft palate (11.0%), urinary system abnormality (hypospadias, renal insufficiency, horseshoe kidney, and uronephrosis) (9.8%), skin disease (eczema, vitiligo, and atopic dermatitis) (6.7%), and short neck (5.5%); 2) psychoneurological abnormalities including intellectual disability (57.1%), language and motor development delay (49.7%), hypotonia (40.5%), delayed myelination (10.4%), and autism (6.7%); and 3) IgA deficiency (4.3%) and hypothyroidism (3.7%).

### Summaries of Karyotypes

**Figure 3:**
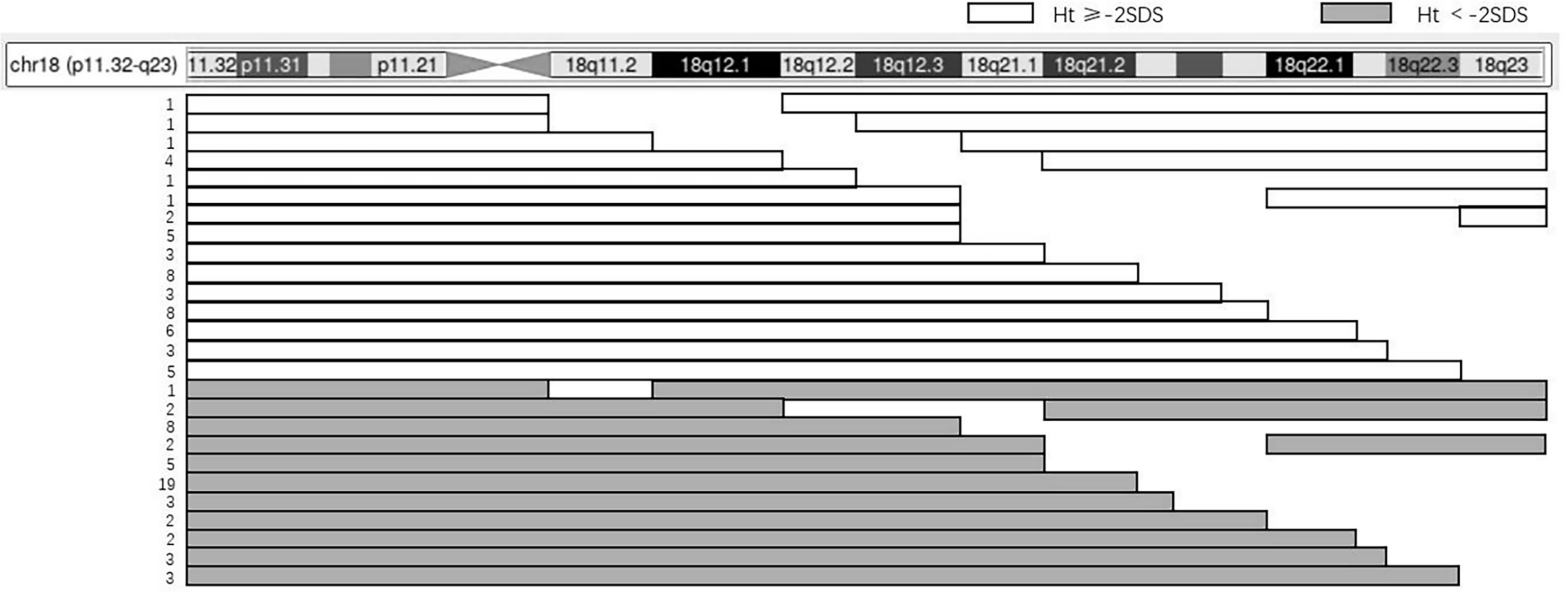
The relation between karyotype and height of 119 patients. The panel illustrates the chromosome content for 119 patients with an 18q deletion and short stature using the UCSC Genome Browser Custom Tracks feature. The horizontal bars depict the region of chromosome 18, while the gaps indicate the hemizygous region. The number of patients that have the same karyotype is shown on the left.

There were 119 cases reported with detailed karyotype descriptions, including 104 cases of terminal fragment deletions and 15 cases of interstitial deletions of the long arm of chromosome 18 (**Figure 3**). The chromosome fragment deletions mainly occurred in the 18q23 (87.4%), followed by the 18q22.3–q23 (84.9%). As for cases with short stature, the chromosome fragment deletions mainly occurred in the 18q23 (90.0%), followed by the 18q22.3–q23 (84.0%).

### Recombinant Human Growth Hormone Treatment in Patients With 18q- Syndrome

Twenty-two patients with 18q- syndrome in the literature and our present case received rhGH treatment. Six cases were excluded from the analysis because of missing treatment information. Thus, a total of 16 cases had been summarized in the study. As shown in **Table 1**, the average age of initial treatment was 3.70 ± 2.90 years, the average treatment duration was 5.90 ± 3.30 years, and the average height SDS was significantly increased from −3.12 ± 0.94 to −1.38 ± 1.29 (p < 0.0001) (**Figure S1**). Among these cases, 13 were reported from the University of Texas Health Science Center. The average dose of rhGH was 0.3 mg/kg/week, the average treatment duration was 6.00 ± 3.20 years, and the average height SDS increase was 1.80 (9).

**Table 1:** Summary of the relevant literature on rhGH treatment for short stature of 18q- syndrome.

Reference	Gender	Age	Dose	Duration (years)	Height SDS before treatment	Height SDS after treatment	ΔHT SDS
Our center	F	93m	0.06 mg/kg/day	7	−3.02	-0.20	2.82
Cody, J D (9)	NA	13m	0.30 mg/kg/week	6.00 ± 3.20	−3.11	+0.85	3.96
	NA	35m	0.30 mg/kg/week		−3.58	−1.55	2.03
	NA	33m	0.30 mg/kg/week		−2.16	+0.45	2.61
	NA	81m	0.30 mg/kg/week		−4.79	−2.15	2.64
	NA	35m	0.30 mg/kg/week		−2.37	−1.10	1.27
	NA	20m	0.30 mg/kg/week		−2.21	−0.65	1.56
	NA	31m	0.30 mg/kg/week		−4.11	−2.50	1.61
	NA	56m	0.30 mg/kg/week		−2.05	−2.06	−0.01
	NA	46m	0.30 mg/kg/week		−1.66	−1.66	0
	NA	64m	0.30 mg/kg/week		−2.59	−0.30	2.29
	NA	5m	0.30 mg/kg/week		−4.20	−3.05	1.15
	NA	9m	0.30 mg/kg/week		−2.60	−1.70	0.90
	NA	31m	0.30 mg/kg/week		−3.30	−0.40	2.90
Jackowski, T (10)	F	20m	0.30 mg/kg/day	8	−4.12	−2.03	2.09
Schwarz, H (7)	F	136m	2.50 mg/qod	1	−4.02	−3.98	0.04
Mean		3.70 ± 2.90 years		5.90 ± 3.30 years	−3.12 ± 0.94	−1.38 ± 1.29	1.74 ± 1.15

NA, not available; rhGH, recombinant human growth hormone; SDS, SD score; HT, height; m, months.

## Discussion

Here, we reported one case of 18q- syndrome successfully treated with long-term rhGH supplement. All previously reported cases in the literature were also reviewed to investigate the karyotype–phenotype relationship and their therapeutic response to rhGH. The phenotype of 18q- syndrome was highly variable characterized by short stature, intellectual disability, and multisystem organ involvement. The literature review suggested a significant height benefit for short stature with 18q- syndrome from long-term rhGH treatment.

The clinical manifestations of 18q- syndrome were complex and diverse, and it was difficult to establish an accurate relationship between genotype and phenotype. There were two main concerns in the karyotype–phenotype relationship: 1) no consistency between the deletion and phenotype had been found in this review of data. The most common cause of the syndrome was the terminal deletion of the long arm of chromosome 18 (87.4%), and the interstitial deletion was rare (12.6%). We compared the clinical characteristics of patients with interstitial deletion and terminal deletion and found that no significant difference existed between these two groups of patients, which was consistent with a previous report (11). 2) There was no absolute correlation between the size of deletions and phenotype, which was also consistent with a previous report (12). A case with 21.77-Mb deletion (18q21.32–q23) was characterized by a range of physical deformities including microcephaly, middle facial dysplasia, cleft lip and palate, slender fingers, atrial septal defect and moderate pulmonary artery stenosis, intellectual disability, intellectual disability, otitis media, and eczema (10). Another case with 34.47-Mb deletion (18q21.1–q23) presented with fewer physical deformities (12). Differences of phenotypes had been reported in family members with the same karyotype with 18q- syndrome (13). However, Kline et al. established a correlation between the size of deletion and clinical severity by array comparative genomic hybridization (CGH) (14). Further investigations are needed to confirm this correlation in a larger cohort. Further studies are also needed to evaluate the important genes that may be involved in the pathogenesis, including SS18 gene (15), CYB5A gene (16), and Sall3 or Tshz1 gene (17), which play key roles in long bone development, gonadogenesis, and development of skull and midline structures.

Short stature is a common issue in the management of 18q- syndrome. Of the reported cases, 35.0% had short stature. There are many reasons for short stature in patients with 18q- syndrome. First, GHD is the main concern in previously reported cases with short stature (18). Our case had prominent short stature with no GHD. More data are needed to assess the status of growth hormone secretion in this group of rare diseases. Second, hypothyroidism is an important contributing factor in short stature. Cases with hypothyroidism had prominent short stature (mean height –2.90 ± 0.80). Shaub et al. reported that the critical region for hypothyroidism was located at the terminal of 18q with the deletion of 13.3 Mb (19). Thyroid hormones are key regulators of bone homeostasis and skeletal development (20). Routine screening for thyroid function could be very beneficial for the management of 18- syndrome, and that thyroid hormone supplementation should be promptly administered to 18q- syndrome with hypothyroidism. Thirdly, important genes on chromosome 18 are effectively involved in the pathogenesis of short stature. In 2009, Cody et al. found that the crucial area was located at 18q22.3–q23 for patients with short stature (21). The myelin basic protein (MBP) gene and the galanin receptor 1 (GALR1) gene are located at 18q23 and are considered to be the main candidate genes for short stature (22). The lack of 18q23 may lead to haplotype insufficiency of *MBP*. *MBP* is the main component of myelin in the central nervous system. Myelin development disorders are considered to have the same critical area as short stature. The propane receptor is a G protein-coupled receptor, ligand of propane is a neuromodulator that is expressed in the central and peripheral nervous system, and it stimulates GH secretion by interacting with specific membrane receptors. But not all missing genes have a phenotype of short stature when hemizygous, and perhaps environmental factors or genes on other chromosomal regions are modulating the phenotype.

The data of rhGH treatment in 18q- patients are limited. In our patient, long-term rhGH treatment successfully increased height SDS, and no obvious side effects had been found. The height of all 16 patients we reviewed had increased by 1.78 SDS after an average treatment duration of 5.90 years. In addition, previous studies have demonstrated that GH treatment had beneficial effects on performance IQ and myelination (9, 22). Multicenter registration researches are needed to further confirm the safety and efficacy of rhGH in the treatment of this rare syndrome.

However, there are potential limitations in this study. First, the sample size was relatively small. Additionally, the lack of detailed information on previously reported cases is a further limitation. Further research is still needed in the future. Another limitation is the lack of assessment and the evolution of diagnostic criteria for GHD.

## Conclusion

The clinical phenotypes of 18q- syndrome are highly variable. Routine physical examination, laboratory investigations, and chromosome screening may facilitate the early diagnosis and treatment of the disease. In addition, the literature review suggested that long-term treatment of growth hormone is effective, and further research is still needed in the future.

## Patient’s Perspective

The patient: “After rhGH treatment, my appetite slightly increased and my height successfully improved, I’m very satisfied”.

## Data Availability Statement

The raw data supporting the conclusions of this article will be made available by the authors, without undue reservation.

## Ethics Statement

The studies involving human participants were reviewed and approved by the ethics committee of the Peking Union Medical College Hospital. Written informed consent to participate in this study was provided by the participants’ legal guardian/next of kin.

## Author Contributions

HP and HZ designed the study. SL collected the data. SL and MC drafted the manuscript. HY guided the writing of the article. HY, SC, LW, LD, HZ, and HP interpreted the data and revised the manuscript. All authors contributed to the article and approved the submitted version.

## Funding

This study was supported by CAMS Innovation Fund for Medical Science (CIFMS-2016-I2M-1-008) and the National Natural Science Foundation of China (No. 81673184).

## Conflict of Interest

The authors declare that the research was conducted in the absence of any commercial or financial relationships that could be construed as a potential conflict of interest.

## Publisher’s Note

All claims expressed in this article are solely those of the authors and do not necessarily represent those of their affiliated organizations, or those of the publisher, the editors and the reviewers. Any product that may be evaluated in this article, or claim that may be made by its manufacturer, is not guaranteed or endorsed by the publisher.
