# Supplementary material for: Clinical Characteristics and Long-Term Recombinant Human Growth Hormone Treatment of 18q- Syndrome: A Case Report and Literature Review
Source: Front Endocrinol (Lausanne). 2021 Dec 9;12:776835. doi: 10.3389/fendo.2021.776835 (PMC8695685; doi:10.3389/fendo.2021.776835)

**Clinical characteristics and long-term rhGH treatment of 18q- syndrome: a case report and literature review**

**Figure S1.** The variables of height SDS between before and after rhGH treatment. *** p＜0.0001


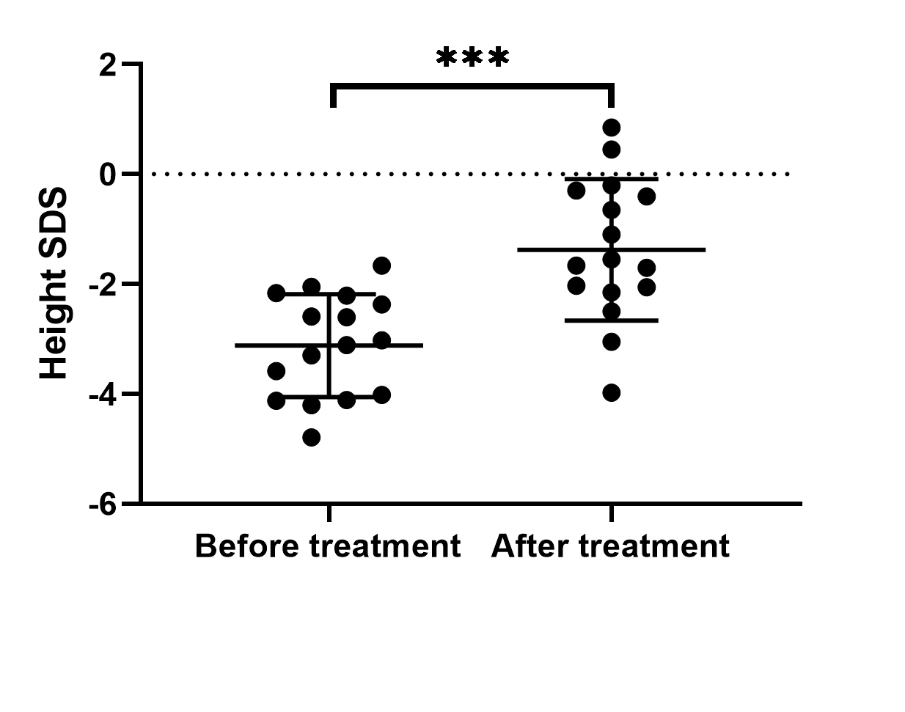

Supplement: Supplementary file 1 [file DataSheet_1.docx]
